# Neonatal White Matter Abnormalities an Important Predictor of Neurocognitive Outcome for Very Preterm Children

**DOI:** 10.1371/journal.pone.0051879

**Published:** 2012-12-28

**Authors:** Lianne J. Woodward, Caron A. C. Clark, Samudragupta Bora, Terrie E. Inder

**Affiliations:** 1 Canterbury Child Development Research Group, Department of Psychology, University of Canterbury, Christchurch, New Zealand; 2 New Zealand Brain Research Institute, Christchurch, New Zealand; 3 Department of Psychology, University of Oregon, Eugene, Oregon, United States of America; 4 Departments of Pediatrics, Neurology and Radiology, Washington University School of Medicine, St Louis, Missouri, United States of America; Hôpital Robert Debré, France

## Abstract

**Background:**

Cerebral white matter abnormalities on term MRI are a strong predictor of motor disability in children born very preterm. However, their contribution to cognitive impairment is less certain.

**Objective:**

Examine relationships between the presence and severity of cerebral white matter abnormalities on neonatal MRI and a range of neurocognitive outcomes assessed at ages 4 and 6 years.

**Design/Methods:**

The study sample consisted of a regionally representative cohort of 104 very preterm (≤32 weeks gestation) infants born from 1998–2000 and a comparison group of 107 full-term infants. At term equivalent, all preterm infants underwent a structural MRI scan that was analyzed qualitatively for the presence and severity of cerebral white matter abnormalities, including cysts, signal abnormalities, loss of white matter volume, ventriculomegaly, and corpus callosal thinning/myelination. At corrected ages 4 and 6 years, all children underwent a comprehensive neurodevelopmental assessment that included measures of general intellectual ability, language development, and executive functioning.

**Results:**

At 4 and 6 years, very preterm children without cerebral white matter abnormalities showed no apparent neurocognitive impairments relative to their full-term peers on any of the domain specific measures of intelligence, language, and executive functioning. In contrast, children born very preterm with mild and moderate-to-severe white matter abnormalities were characterized by performance impairments across all measures and time points, with more severe cerebral abnormalities being associated with increased risks of cognitive impairment. These associations persisted after adjustment for gender, neonatal medical risk factors, and family social risk.

**Conclusions:**

Findings highlight the importance of cerebral white matter connectivity for later intact cognitive functioning amongst children born very preterm. Preterm born children without cerebral white matter abnormalities on their term MRI appear to be spared many of the cognitive impairments commonly associated with preterm birth. Further follow-up will be important to assess whether this finding persists into the school years.

## Introduction

Central to reducing the longer-term morbidities associated with very preterm birth is the need to identify early neonatal markers of risk. Children born very preterm now represent almost 2% of all live births in the U.S. and other developed countries, [1] with follow-up studies identifying high rates of subsequent neurodevelopmental impairment. Approximately 10% of survivors develop neurosensory impairments such as cerebral palsy, blindness, and deafness. A further 40% will be affected by cognitive deficits, language problems, developmental coordination disorder, inattention, and educational underachievement. [2], [3], [4], [5] These impairments pose major challenges, with a third of children born very preterm requiring ongoing specialist health care and up to two-thirds needing educational or psychological support during their school years. [6], [7], [8].

To date, efforts to identify early perinatal markers of later neurodevelopmental risk have been somewhat disappointing, with most factors correlating only modestly with outcome. One potential exception is the presence and severity of cerebral white matter abnormalities on term magnetic resonance imaging (MRI). These diffuse white matter abnormalities affect between 50–70% of very preterm infants and include: white matter volume loss; ventriculomegaly; white matter signal abnormality; thinning of the corpus callosum; and delayed myelination. [9], [10] White matter abnormalities have been shown to be more predictive of later neurodevelopmental impairment than gestational age and other perinatal factors. [11], [12], [13], [14] Short-term follow-up studies suggest that prediction may be better for motor than for cognitive outcomes. [12], [14] However, a recent study of 60 very preterm born children at age 9, found that moderate-to-severe white matter abnormalities on term MRI were associated with increased risks of both cognitive delay (WISC score <85; Odds ratio, OR = 8) and cerebral palsy (OR = 10), with these associations persisting after adjustment for other clinical risk factors. [11].

However, some caution is warranted. First, with the exception of Iwata, [11] follow-up beyond 2 to 3 years is extremely rare. [15] Given the extended developmental trajectories of many cognitive skills, longer-term follow-up is essential to provide an accurate assessment of clinically important neurocognitive impairments. This need is further reinforced by findings that early measures, such as the Bayley Scales, correlate only modestly with longer-term cognitive outcome. [16], [17] A second limitation of existing research has been the reliance on a single, typically global, measure of cognition making it difficult to assess whether associations are pervasive or confined to specific cognitive domains. Third, considerable variability exists with respect to the measurement of white matter abnormalities, with some studies grouping children with no and mild abnormalities [12], [18] and others combining all white matter abnormalities cases. [11] These differences make comparison across studies difficult and also potentially obscure linear relationships in the data. [13], [14] Fourth, there is a critical need for comparative studies to include a representative group of full-term born children. Recent difficulties with the Bayley-III norms underscore this point. [19], [20] Finally and importantly, given the strong influence of family factors such as maternal education and socioeconomic status on child development, it is essential that such factors also be taken into account. To date, such factors have been largely ignored in neuroimaging outcome studies of children born very preterm.

To address these limitations, the present study draws on prospective longitudinal data from a representative cohort of children born very preterm and a comparison group of their full-term peers to assess the cognitive, language, and executive functioning outcomes of very preterm born children from 4 to 6 years. In particular, we examined the extent to which neonatal white matter abnormalities on MRI were predictive of later impairment across a range of neurocognitive outcomes assessed at multiple time points, thus, allowing for an assessment of the stability of associations across time/age. We hypothesized that increasing severity of white matter abnormalities at term equivalent would be associated with increasing risks of impairment across all outcomes/ages, with these associations persisting after taking into account other medical and social risk factors.

## Methods

### Participants

Study participants consisted of two groups of children. The first group was a regional cohort of 110 children born very preterm (≤32 weeks gestation) who were consecutively admitted to a level III Neonatal Intensive Care Unit (NICU) at Christchurch Women’s Hospital (New Zealand) over a two-year period (November 1998–December 2000). In total, 92% of eligible infants were recruited. Excluding deaths (*n* = 3), 98% were followed up at age 4 and 97% at age 6. In addition, one child was excluded due to blindness and another due to missing MRI data, leaving a final sample of 104 children at age 4 and 102 children at age 6.

The second group of study children, recruited at age 2 years, comprised a sample of 113 full-term born (37–41 weeks gestation) children. These children were identified from hospital birth records for the same period by alternately selecting, in a forwards and backwards fashion, the second child listed in the delivery schedule. As a group, they were matched to the very preterm cohort for gender, delivery hospital, and date of birth. Of those identified, 62% were recruited at age 2. Reasons for nonparticipation included untraced (*n* = 32), moved overseas (*n* = 9), refusals (*n* = 9), and agreed but couldn’t attend clinic appointment/s within the 2-week assessment window due to illness or family circumstances (*n* = 19). No significant differences were found between recruited and nonrecruited term born infants on measures of gestational age, birth weight, socioeconomic status, or race. In addition, comparison of the socioeconomic profile of recruited families with regional census data [21] showed that they were highly representative of the Canterbury region from which they were recruited. Retention to ages 4 and 6 years was 96% (*n* = 108). Data for one additional child was excluded due to incompleteness. Written informed consent was obtained from all parents/guardians and all procedures were approved by our regional Health and Disability Ethics Committee. Table 1 describes the infant clinical and family background characteristics of the two study groups.

**Table 1:** Clinical and Social Background Characteristics of the Sample.

Measure	Full-Term (*N* = 109)	Very Preterm (*N* = 106)	*p*
**Infant Clinical Characteristics**			
Gestational age, *M* ± *SD*, weeks	39.5±1.2	27.9±2.3	<.0001
Birth weight, *M* ± *SD*, grams	3,574.6±407.9	1,065.9±312.6	<.0001
Male sex, %	54.1	50.9	.64
Twin births, %	3.7	34.0	<.0001
Small for gestational age^a^, %	0.9	10.4	.003
Oxygen therapy at 36 weeks, %	–	34.0	
Antenatal corticosteroid use, %	–	84.0	
Postnatal dexamethasone use, %	–	5.7	
Intraventricular hemorrhage grade III or IV^b^, %	–	5.7	
White matter abnormalities on MRI			
None, %	–	24.8	
Mild, %	–	57.1	
Moderate-to-Severe, %	–	18.1	
**Social Background Characteristics**			
Maternal age, *M* ± *SD*, years	31.0±4.5	30.8±5.3	.75
Mother no high school graduation, %	19.3	39.6	.001
Single parenthood, %	11.9	18.9	.16
Minority ethnicity, %	11.9	13.2	.78
Family socioeconomic status^c^			
Professional/managerial, %	35.8	26.4	
Technical/skilled, %	54.1	43.4	
Semiskilled/unskilled/unemployed, %	10.1	30.2	.001

a Birth weight more than 2 standard deviations below the mean for gestational age and sex.

b Based on Papile classification.

c Assessed using the Elley-Irving Socioeconomic Index (Elley & Irving, 2003).

### Measures

#### MRI scanning (Term equivalent)

All very preterm born children underwent structural MR imaging at term equivalent age (39–41 weeks gestation). Unsedated infants were settled, wrapped, and placed in a Vac Fix bean bag and then scanned using a 1.5-tesla General Electric Signa System (GE Medical Systems) with previously documented sequences. [10] Each infant’s scan was then graded using a qualitative scoring system consisting of five 3-point scales assessing the presence and severity of periventricular white matter volume loss, white matter signal abnormality, the presence of cystic abnormalities, ventricular dilation, and thinning of the corpus callosum and reduced myelination (see supplement to [14]). All scans were scored by a blinded pediatric neuroradiologist and independently reviewed by a pediatric neurologist. Inter-rater agreement was 95%, with discrepant cases given a consensus rating. Based on their total white matter abnormality scores, children were classified as follows: 1) no abnormalities (score of 5 to 6, *n* = 26/106); 2) mild abnormalities (score of 7 to 9, *n* = 61/106); 3) moderate abnormalities (score of 10 to12, *n* = 16/106); or 4) severe abnormalities (score >12, *n* = 3/106). Given the small number of children with severe white matter abnormalities, children in the moderate and severe white matter abnormalities groups were combined for the purposes of this analysis.

#### Neurocognitive outcomes at ages 4 and 6 years

At corrected ages 4 (±2 weeks) and 6 (±4 weeks), all children underwent a comprehensive multidisciplinary assessment of their general cognitive ability (IQ), language development, and executive functioning skills. At age 4, independent testers administered each measure, while at 6, one administered the IQ and language measures and another administered the executive functioning test battery. All assessments were done blind to child perinatal history and past cognitive performance. Clinical impairment was defined on the basis of the score distributions of our regionally representative comparison group. [2], [22] This approach minimizes problems associated with the use of outdated test norms due to secular changes in test scores over time. [23], [24] Mild delay was defined by a score that was more than 1 standard deviation (*SD*) below the comparison group mean and severe delay was defined by a score that was more than 2 standard deviations below the comparison group mean. Study measures included in this analysis were as follows.

#### Cognitive ability

At ages 4 and 6 (corrected), children’s intellectual ability was assessed using a short form of the revised Wechsler Preschool and Primary Scales of Intelligence (WPPSI-R) [25] consisting of two verbal (Comprehension and Arithmetic) and two performance (Picture Completion and Block Design) subtests. This measure has been shown to be reliable and to correlate highly with the full scale WPPSI (*r* = .89–.92). [26].

#### Language development

At age 4 (corrected), the preschool version of the Clinical Evaluation of Language Fundamentals (CELF-P) [27] was used to provide a standardized measure of expressive and receptive language ability. The CELF-P consists of six subtests assessing Linguistic Concepts, Basic Concepts, Sentence Structure, Recalling Sentences in Context, Formulating Labels, and Word Structure. Children’s performance across these subtests was summed to provide an overall measure of both receptive and expressive language development. This scale is internally consistent, has good test-retest reliability and correlates well (*r* = .90) with other language measures. [28] At age 6, the Understanding Directions subtest from the Woodcock Johnson-III Tests of Achievement (WJ-III) [29] was used to measure child language at school age. This subtest assesses receptive language by requiring children to comprehend and respond to sequences of increasingly complex verbal instructions. The WJ-III is an established measure of school achievement and has excellent psychometric properties, including good test-retest reliability (0.7–0.9).

#### Executive functioning

Children’s executive function abilities were assessed using a battery of age appropriate tasks administered at both follow-up time points. At age 4, this battery consisted of four tasks: 1) Tower of Hanoi (planning and problem solving) [30]; 2) Flexible Item Selection (cognitive flexibility) [31]; 3) Visual Search (selective attention) [30]; and 4) the Shape School task (inhibition and cognitive flexibility) [32]. At age 6, this battery consisted of: 1) Tower of Hanoi (planning and problem solving) [30]; 2) Visual Search (selective attention) [33]; 3) Backward Digit Span (verbal working memory) [34]; 4) Backward Corsi Blocks (visuospatial working memory) [35]; 5) Detour Reaching Box (inhibition, cognitive flexibility) [36]; and the 6) Conners’ Kiddie-Continuous Performance Test (inhibitory control and sustained attention) [37]. As described previously, [38] a composite measure of executive function performance was computed for each follow-up, with scores standardized to a mean of 10 and standard deviation of 2 to allow direct comparison across time points. These composite scores were created on the basis of exploratory and confirmatory factor analyses showing that individual tasks loaded onto a single common factor, with loadings exceeding.6 and communalities being acceptable (>.4).

## Results

### Neurocognitive Outcomes of Very Preterm and Full-Term Children

At corrected ages 4 and 6 years, very preterm born children performed less well than full-term born children across all measures of intellectual ability, language, and executive functioning. As shown in Table 2, at age 4, the mean difference between very preterm and full-term children’s IQ scores on the WPPSI-R was 0.7 of a standard deviation with this gap increasing to 0.9 by age 6. Mean differences for language and executive function were more stable at around 0.5 of a standard deviation at both ages.

**Table 2:** Neurocognitive Outcomes of Very Preterm and Full-Term Children at Ages 4 and 6 Years.

Measure	Full-Term (*N* = 109)	Very Preterm (*N* = 106)	t/χ^2^	*P*
**Age 4 Years**				
**Intellectual Ability**	(*n = *107)	(*n = *104)		
Total IQ score, *M* ± *SD*	104.7±13.4	94.9±15.7	4.89	<.0001
Any delay, %	13.1	34.6	13.52	<.0001
Severe delay, %	1.9	9.6	5.90	.02
**Language Development**	(*n = *104)	(*n = *99)		
CELF Score, *M* ± *SD*	98.2±13.4	90.5±14.3	3.95	<.0001
Any delay, %	15.4	31.3	7.23	.007
Severe delay, %	1.9	8.1	4.11	.05
**Executive Functioning**	(*n = *105)	(*n = *103)		
Total score, *M* ± *SD*	10.5±1.9	9.5±2.0	3.89	<.0001
Any delay, %	17.1	32.0	6.23	.01
Severe delay, %	2.9	9.7	4.17	.04
**Age 6 Years**				
**Intellectual Ability**	(*n* = 108)	(*n = *102)		
Total IQ score, *M* ± *SD*	106.9±11.7	94.9±15.6	6.34	<.0001
Any delay, %	16.7	49.0	25.08	<.0001
Severe delay, %	3.7	15.7	8.74	.003
**Language Development**	(*n* = 107)	(*n = *100)		
Understanding Directions score, *M* ± *SD*	113.5±7.9	109.2±11.5	3.18	.002
Any delay, %	15.0	30.0	6.77	.009
Severe delay, %	2.8	9.0	3.63	.06
**Executive Functioning**	(*n* = 108)	(*n = *102)		
Total score, *M* ± *SD*	10.7±1.7	9.3±2.1	5.42	<.0001
Any delay, %	15.7	41.2	16.80	<.0001
Severe delay, %	5.6	21.6	11.64	.001

With respect to risks of delay, at age 4, very preterm born children were 2.6 times more likely to be classified as having any cognitive delay (35% v. 13%) and 5 times more likely to be severely delayed (10% v. 2%) than full-term children. Relative risks at age 6 were generally similar, with closer to half of very preterm born children showing any delay (49% v. 17%) and around 1 in 6 severe cognitive delay (16% v. 4%). Fairly consistent rates of language delay were evident, with very preterm born children being twice (31% v. 15%) as likely as full-term children to have any delay and 3–4 times (9% v. 3%) more likely to have severe delay at ages 4 and 6 years. Finally, in terms of performance on the two executive task batteries, at 4 years, very preterm born children were almost twice as likely to show any delay (>1 *SD* below control mean) and 3.3 times more likely to meet criteria for severe delay/impairment (>2 *SD* below control mean). By age 6, similar to intelligence, risks of executive function delay/impairment in the very preterm born group had tended to increase, with relative risks for any delay being 2.6 (41% v. 16%) and for severe delay being closer to 4 (22% v. 6%).

### Relationship between Cerebral White Matter Abnormalities on Term MRI and Later Neurocognitive Outcomes

Table 3 describes the performance of very preterm born children with varying degrees of cerebral white matter abnormalities on their term MRI (none, mild, moderate-to-severe) relative to their full-term peers on measures of intelligence, language, and executive functioning at ages 4 and 6 years. Across both follow-up assessments, clear linear associations were found between the severity of white matter abnormalities on term MRI and children’s later cognitive scores as well as their risks of both any (>1 *SD* below control mean) and severe delay (>2 *SD* below control mean) on measures of intelligence (*p*<.0001), language (*p*≤.003), and executive functioning (*p*<.0001). Specifically, children in the full-term control group obtained the highest scores and had the lowest risk of any or severe delay. In contrast, across the three very preterm born groups, as the severity of white matter abnormalities increased there were corresponding declines in children’s mean scores and risks of clinically significant delay at ages 4 and 6.

**Table 3:** Neurocognitive Outcomes of Very Preterm Children with None, Mild, and Moderate-to-Severe White Matter Abnormalities (WMA) on Neonatal MRI Compared to Full-Term Children.

Measure	Full-Term (FT)	Very Preterm (VPT)			*p* values			
		None WMA	Mild WMA	Moderate-to-Severe WMA	Overall	FT v. VPT None WMA	FT v. VPT Mild WMA	FT v. VPT Moderate-to-Severe WMA
**Age 4 years**								
**Intellectual Ability**	(*n = *107)	(*n* = 25)	(*n* = 60)	(*n* = 19)				
IQ score, *M* ± *SD*	104.7±13.4	102.2±17.5	96.5±12.1	80.3±14.5	<.0001	.41	<.0001	<.0001
Any delay, %	13.1	16.0	30.0	73.7	<.0001	.70	.009	<.0001
Severe delay, %	1.9	4.0	5.0	31.6	<.0001	.53	.27	<.0001
**Language Development**	(*n = *104)	(*n* = 22)	(*n* = 58)	(*n* = 19)				
CELF score, *M* ± *SD*	98.2±13.4	94.6±16.6	92.5±11.5	79.5±14.5	<.0001	.25	.01	<.0001
Any delay, %	15.4	18.2	25.9	63.2	<.0001	.74	.11	<.0001
Severe delay, %	1.9	4.5	3.4	26.3	.002	.48	.55	.001
**Executive Functioning**	(*n = *105)	(*n* = 24)	(*n* = 60)	(*n* = 19)				
Total score, *M* ± *SD*	10.5±1.9	10.4±1.5	9.5±1.9	8.1±2.4	<.0001	.74	.002	<.0001
Any delay, %	17.1	12.5	31.7	57.9	<.0001	.58	.03	<.0001
Severe delay, %	2.9	0	6.7	31.6	<.0001	–	.26	<.0001
**Age 6 years**								
**Intellectual Ability**	(*n = *108)	(*n* = 22)	(*n* = 60)	(*n* = 19)				
Total score, *M* ± *SD*	106.9±11.7	101.7±13.1	96.4±12.6	82.1±19.9	<.0001	.09	<.0001	<.0001
Any delay, %	16.7	22.7	51.7	73.7	<.0001	.50	<.0001	<.0001
Severe delay, %	3.7	9.1	11.7	36.8	<.0001	.29	.06	<.0001
**Language Development**	(*n = *107)	(*n* = 22)	(*n* = 59)	(*n* = 18)				
Understanding directions, *M* ± *SD*	113.5±7.9	113.7±8.2	110.3±8.2	100.1±18.1	<.0001	.91	.04	<.0001
Any delay, %	15.0	9.1	30.5	50.0	<.0001	.48	.02	.001
Severe delay, %	2.8	4.5	5.1	27.8	.003	.67	.46	.001
**Executive Functioning**	(*n = *108)	(*n* = 22)	(*n* = 60)	(*n* = 19)				
Total score, *M* ± *SD*	10.7±1.7	9.9±2.1	9.4±1.9	8.2±2.3	<.0001	.08	<.0001	<.0001
Any delay, %	15.7	27.3	40.0	63.2	<.0001	.20	.001	<.0001
Severe delay, %	5.6	18.2	20.0	31.6	<.0001	.06	.006	.001

Closer examination of group contrasts showed the following:

1. Relative to the full-term group, very preterm born children with moderate-to-severe neonatal white matter abnormalities had the most impaired neurocognitive performance. At ages 4 and 6, they were 3.3–5.6 times more likely to meet criteria for any delay and 3.4–16.6 times more likely to be subject to severe delay. Specifically, 74% had cognitive delay at ages 4 and 6, 63% had language delay at age 4 and 50% at age 6, and 58–63% showed impaired performance on the executive function task battery. Risks of severe intellectual (32–37%), language (26–28%), and executive functioning (32%) impairment were also high and largely confined to this group.
2. Very preterm born children with mild white matter abnormalities on neonatal MRI were, in general, at lower risk of adverse neurocognitive outcomes than very preterm children with moderate-to-severe white matter abnormalities, and at higher risk than very preterm born children without white matter abnormalities and full-term children. Compared to the full-term group, they were 1.7–3.0 times more likely to show any delay and 1.8–3.6 times more likely to exhibit severe delay on measures of IQ, language, and executive functioning. Most of the problems in this group were in the mild (26–52%) as opposed to the severe (3–20%) range.
3. No significant differences were found between very preterm born children without white matter abnormalities and full-term comparison children on any of the outcome measures assessed at either 4 or 6 years (*p*>.05). Mean score differences were generally small, as were relative risks for any (RR = 1.2–1.7) and severe delay (RR = 1.6–3.2).

Extending on these findings, linear associations between the individual MRI scale scores used to assess white matter abnormalities were examined in relation to each outcome. A generally consistent pattern of findings emerged across measures, although associations tended to be stronger at age 4 than at age 6. White matter signal abnormality (*r* = -.44 at age 4 and -.29 at age 6, *p*≤.003), reduced white matter volume (*r* = -.42 and -.37, *p*<.0001), cystic abnormality (*r* = -.35 and -.24, *p*≤.02), ventricular dilation (*r* = -.26 and -.28, *p*≤.007) and thinning of the corpus callosum/reduced myelination (*r* = -.24 and -.28, *p*≤.02) were all associated with IQ. White matter signal abnormality (*r* = -.31 and -.32, *p*≤.002), reduced white matter volume (*r* = -.31 and -.36, *p*≤.002), thinning of the corpus callosum/reduced myelination (*r* = -.24 and -.30, *p*≤.02) and cystic abnormality (*r* = -.30 and -.38, *p*≤.002) were also correlated with later language function. Finally, a similar, but generally weaker, pattern of associations was evident for executive functioning at ages 4 and 6 (*r* = -.17 to -.31 at 4 and *r* = -.09 to -.25, at 6).

### Other Medical and Social Background Factors Associated with Child Neurocognitive Outcome

To examine the extent to which other infant medical and family factors might also place very preterm born children at elevated risk of later neurocognitive impairment, bivariate associations between each outcome and a wide range of infant perinatal, maternal and family social background factors were examined. These analyses were conducted based on the very preterm born group and/or the total sample where appropriate. Infant medical characteristics examined included gestational age at birth, birth weight, gender, small for gestational age, antenatal corticosteroid use, postnatal dexamethasone exposure, chronic lung disease, patent ductus arteriosus, necrotizing enterocolitis, and confirmed sepsis. Family social background factors included maternal age at birth, maternal pregnancy smoking, family socioeconomic status, maternal education, family type (two or one parent), and race.

Across these analyses, few medical risk factors were correlated with outcome. Antenatal corticosteroid exposure in very preterm born children appeared to be protective for general cognitive delay at both ages 4 (OR = 0.3, *p*<.05) and 6 (OR = 0.3, *p*<.05) whereas being born small for gestational age increased risks of cognitive delay at age 6 (OR = 4.6, *p* = .01). A more consistent and stronger pattern of associations emerged for social risk factors, with lower family socioeconomic status (OR = 2.2–2.8, *p*<.05), low maternal education (OR = 2.8, *p = *.002), and being born to a single mother (OR = 2.5, *p = *.02) placing children at increased neurocognitive risk. Examination of the cumulative effects of these social risk factors showed that each additional risk factor increased the likelihood of an adverse cognitive outcome by 1.7 fold at age 4 (*p* = . 01)and 1.6 at age 6 (*p* = .003).

### Cerebral White Matter Abnormalities on Term MRI and Later Risk of Neurocognitive Delay after Adjustment for Other Medical and Family Factors

To examine the extent to which the presence and severity of cerebral white matter abnormalities on term MRI made an independent net contribution to the prediction of subsequent cognitive risks at ages 4 and 6 years, the associations shown in Table 3 were adjusted for the effects of the medical and social risk factors listed above using logistic regression analysis. Across all outcomes, these analyses were done in two ways. First, all covariates listed above were entered individually. Second, composite measures of neonatal medical and social risk were entered alongside gender. The medical risk index (scaled 0–7) consisted of the sum of the following dichotomous variables: small for gestational age; oxygen therapy at 36 weeks; postnatal dexamethasone use; necrotizing enterocolitis; patent ductus arteriosus; retinopathy of prematurity; and confirmed sepsis. The social risk index (scaled 0–5) consisted of the sum of the following dichotomous variables: early motherhood (<21 years); low maternal education (not a high school graduate); single parent family; low family socioeconomic status (semiskilled, unskilled, or unemployed); and minority race/ethnicity. Both of these analyses produced an identical set of results. Thus Table 4 shows the unadjusted and adjusted odds of later cognitive, language, and executive delay for very preterm children in each of the white matter abnormality groups relative to their full-term peers after adjustment for the effects of gender and the two composite medical and social risk indices. With the exception of executive function delay at age 6 (*p = *.07), cerebral white matter abnormalities remained a significant independent predictor of neurocognitive outcome across all measures. The odds of later cognitive, language, and executive function delay for very preterm children with mild white matter abnormalities were reduced from 1.9–5.3 to 1.8–4.0, while for very preterm children with moderate-to-severe white matter abnormalities they were reduced from 5.7–18.6 to 4.5–15.5 after covariate adjustment.

**Table 4:** Unadjusted and Adjusted Odds of Neurocognitive Delay for Very Preterm Children with None, Mild, and Moderate-to-Severe White Matter Abnormalities (WMA) on Neonatal MRI Relative to Children in the Full-Term Group.

Measure	Age 4 Years				Age 6 years			
	Unadjusted OR (95% CI)	*p*	Adjusted^a^ OR (95% CI)	*p*	Unadjusted OR (95% CI)	*p*	Adjusted^a^ OR (95% CI)	*p*
**Any Intellectual Delay**								
None WMA	1.3 (0.4–4.2)		1.1 (0.3–3.9)		1.5 (0.5–4.5)		1.1 (0.4–3.6)	
Mild WMA	2.8 (1.3–6.3)		2.7 (1.0–7.3)		5.3 (2.6–10.9)		4.0 (1.6–9.8)	
Moderate-to-Severe WMA	18.6 (5.8–59.7)	<.0001	15.5 (3.6–66.6)	.002	14.0 (4.5–43.8)	<.0001	8.1 (2.1–31.7)	.003
**Any Language Delay**								
None WMA	1.2 (0.4–4.1)		1.1 (0.3–3.9)		0.6 (0.1–2.7)		0.5 (0.1–2.2)	
Mild WMA	1.9 (0.9–4.2)		1.8 (0.7–4.9)		2.5 (1.2–5.4)		2.4 (0.9–6.1)	
Moderate-to-Severe WMA	9.4 (3.2–27.6)	<.0001	7.8 (1.9–31.4)	.03	5.7 (2.0–16.5)	<.0001	4.5 (1.2–17.7)	.04
**Any Executive Functioning Delay**								
No WMA	0.7 (0.2–2.6)		0.6 (0.2–2.5)		2.0 (0.7–5.9)		1.6 (0.5–5.0)	
Mild WMA	2.2 (1.1–4.7)		2.2 (0.9–5.6)		3.6 (1.7–7.4)		2.8 (1.1–7.1)	
Moderate-to-Severe WMA	6.6 (2.3–18.9)	<.0001	5.3 (1.4–20.4)	.04	9.2 (3.2–26.7)	<.0001	5.3 (1.4–20.2)	.07

a Adjusted for child sex, neonatal medical risk, and family social risk.

## Discussion

Findings confirm that preschool and early school-aged children born very preterm are at high risk of neurocognitive delay and/or impairment, [23], [39], [40] with these risks evident across measures of general intelligence, language, and executive functioning at both 4 and 6 years. By age 4, at least a third of very preterm born children compared to around 15% of full-term children were subject to some form of neurocognitive delay. Mild delay was more common than severe delay. Only 1 in 3 children in the very preterm group who met criteria for delay were severely delayed (>2 *SD* below control mean) compared to just over 1 in 8 of full-term children (2–3% v. 10% overall). At age 6, a similar pattern of results was observed. Although there was some suggestion that the proportion of very preterm children subject to intellectual and/or executive function delay may have increased somewhat, with between 40–50% meeting criteria for any delay and 15–20% severe delay. This small upward trend may reflect tester or measurement effects, or may well suggest a tendency for these domain impairments to become increasingly apparent with age and the transition to school. Further follow-up using repeated measures and analysis methods that take account of measurement error will be important to help clarify this issue.

To date, most follow-up studies in very preterm born children have tended to focus on the risks of severe impairment and/or disability. However, given the substantial numbers of very preterm survivors with mild cognitive impairment, this group appears to be as important. These less severe but clinically significant impairments will impede children’s early school progress and have cumulative impacts on achievement and the need for remedial support. They are also likely to be more responsive to treatment. Therefore, targeting these children during the important preschool and early school years may be more effective strategy to reduce the morbidities associated with very preterm birth than focusing solely on only those with the most severe impairments. Recognition of milder impairments is also consistent with the secular trend for a reduction in major cystic white matter lesions that traditionally have been associated with more severe disability. Instead, an increasing number of children will likely experience milder forms of white matter abnormality that will result in high-incidence, low-severity conditions (e.g., learning disabilities, ADHD, developmental coordination disorder) that compromise classroom learning. [41].

Key to reducing longer term risks associated with very preterm birth is the need for a better understanding of the neuropathological mechanisms that place very preterm born children at neurocognitive risk. This issue was the central focus of this paper. Across all outcome measures assessed at both time points, clear linear relationships were found between the presence and severity of cerebral white matter abnormalities defined qualitatively on neonatal MRI and children’s later risks of both any and severe neurocognitive delay. Specifically, findings suggested that very preterm infants who are spared cerebral white matter abnormalities can be expected to have similar levels of preschool and early school age cognitive functioning as their full-term peers with respect to general intelligence, language, and executive functioning (i.e., working memory, inhibitory control and cognitive flexibility). In contrast, very preterm born children with white matter abnormalities on term MRI were at increased risk of delays, with risks increasing with the severity of neonatal white matter abnormalities. Those with moderate-to-severe white matter abnormalities were at greatest risk of both mild (>−1 *SD* and<−2 *SD*) and severe (>−2 *SD*) delay, and particularly severe delay. Very preterm born children with mild white matter abnormalities also had increased risks of neurocognitive delay relative to their full-term peers, but their difficulties tended to be of a less severe nature.

These findings extend on previous within-group analyses showing that cerebral white matter abnormalities on term MRI are associated with increased risks of severe cognitive delay at age 2 [14] and any delay at age 9 years based on a single global measure. [11] However, with the exception of two studies, [12], [38] the relative risks for very preterm born children with neonatal white matter abnormalities have not been examined in relation to a comparison group of their full-term peers. Of the two studies that do exist, one found that extremely preterm (*N* = 107, <27 weeks gestation) children with no-to-mild and moderate-to-severe white matter abnormalities had lower mean Bayley-III cognitive and language scores than full-term children at age 30 months (*N* = 85). This finding differs somewhat from the present study, which failed to find any differences between very preterm born children with no cerebral white matter abnormalities and full-term control children. This may reflect the high-risk extremely preterm sample and/or the grouping of no and mild white matter abnormality cases together. The second study to include a full-term comparison group focused on a series of domain specific executive functioning outcomes and reported a similar pattern of findings. [38] Since this was based on the same sample as the current study, further replication is clearly needed.

The composite nature of the qualitative rating system used to classify white matter abnormalities makes it difficult to speculate confidently on the neural mechanisms underlying these neurocognitive impairments. Examination of the individual white matter abnormality subscales in relation to neurocognitive outcomes suggested that all were contributing to later risk, but that white matter signal abnormalities and volume loss may be particularly relevant. Very preterm born children with periventricular white matter signal abnormalities have been shown to be characterized by less mature fiber tract development and organization, particularly in those fibers tracking through the internal capsule and nearby regions. [42] Consistent with this, cross-sectional diffusion tensor imaging (DTI) studies of older very preterm children have found links between IQ and reduced fractional anisotropy (FA) values in central and peripheral white matter tracts including inferior and superior fasciculi, thalamocortical and other tracts passing through the internal and external capsule. [43], [44], [45] Reduced white matter volume has also been confirmed by volumetric MRI studies at term and throughout childhood and adolescence [46] and been shown to correlate with intellectual delay and executive functioning [47] even after adjustment for neonatal risk factors. [48] The presence of white matter signal abnormalities and volume loss may reflect more than isolated white matter injury and loss of axons. [49] These white matter abnormalities may be visible markers for more diffuse neuronal abnormalities resulting from the secondary effects of axonal deafferentation as well as the less visible, but equally prominent, direct neuronal and subplate neuronal injury. [50] An association between white matter abnormality and reduction in cortical gray matter volume is consistent with this hypothesis. [51] Alterations in cortical and deep nuclear gray matter neuronal connectivity and functioning, via primary injury or secondary degeneration following axonal deafferentation, may impact subsequent cortical development and function with long term implications for cognition, language, and executive functioning. Currently, visible neuroimaging markers of the neuronal impact of very preterm birth are lacking and would likely compliment the current white matter abnormality evaluation. Although this should be the focus of future research, the current study does emphasize the importance of cerebral white matter abnormalities in relation to the longer term outcomes of the very preterm infant.

The importance of cerebral white matter abnormalities as a predictor of neurocognitive delay for very preterm born children was further reinforced after extensive covariate adjustment for gender, other neonatal medical factors, and family social risk. White matter abnormality severity remained a significant independent predictor of later intellectual, language, and executive function delay. The only exception was executive function delay/impairment at age 6 which was reduced to marginal significance (mild white matter abnormality OR = 2.8, moderate-to-severe white matter abnormality OR = 5.3, *p* = .07). Irrespective of the modeling approach used, consistent with other studies, [11], [48] few significant medical covariates were found. In contrast, social risk factors such as family socioeconomic status, maternal educational under-achievement, and single parent family were found to be important predictors of neurocognitive delay both individually and in combination with white matter abnormality. These findings highlight the importance of the childrearing environment in shaping the cognitive outcomes of very preterm born children. They also suggest that conventional MRI findings, while informative for follow-up and intervention planning, are unlikely on their own to ever be sufficient for accurate risk prediction. Rather, MRI findings need to be considered alongside other health and social factors known to be important for child development.

The strengths of this study include the prospective longitudinal design, the unselected sample of children born very preterm in our region, inclusion of a regionally representative comparison group, assessment of neurocognitive outcome using multiple measures at multiple time points, the use of blinded independent testers, and our high sample recruitment and retention. However, the study was limited by the use of an abbreviated rather than full WPPSI-R at ages 4 and 6 years. This approach was adopted to minimize sample member over-burden and to allow a wider range of measures to be collected. A second limitation was that we did not administer the same language measure at both ages. Receptive and expressive language was assessed at age 4, whereas only receptive skills were assessed at age 6.

Nonetheless, findings indicate that neonatal white matter abnormalities on MRI at term represent an important predictor of persistent neurocognitive problems during the preschool and early school years. Conventional MRI at term equivalent is likely to provide useful information to assist with post discharge planning and monitoring when interpreted alongside other influences on child cognitive development such as family social circumstances and functioning.
